# Unraveling connectivity changes due to dopaminergic therapy in chronically treated Parkinson’s disease patients

**DOI:** 10.1038/s41598-018-31988-0

**Published:** 2018-09-25

**Authors:** Tommaso Ballarini, Filip Růžička, Ondrej Bezdicek, Evžen Růžička, Jan Roth, Arno Villringer, Josef Vymazal, Karsten Mueller, Matthias L. Schroeter, Robert Jech

**Affiliations:** 1 Max-Planck Institute for Human Cognitive and Brain Sciences, Leipzig, Germany; 2 Department of Neurology, Charles University in Prague, First Faculty of Medicine, Prague, Czech Republic; 3 Clinic for Cognitive Neurology, University Clinic, Leipzig, Germany; 4 Department of Radiology, Na Homolce Hospital, Prague, Czech Republic; 5 FTLD Consortium, Ulm, Germany

## Abstract

The effects of dopaminergic therapy for Parkinson’s disease (PD) on the brain functional architecture are still unclear. We investigated this topic in 31 PD patients (disease duration: 11.2 ± (SD) 3.6 years) who underwent clinical and MRI assessments under chronic dopaminergic treatment (duration: 8.3 ± (SD) 4.4 years) and after its withdrawal. Thirty healthy controls were also included. Functional and morphological changes were studied, respectively, with eigenvector centrality mapping and seed-based connectivity, and voxel-based morphometry. Patients off medication, compared to controls, showed increased connectivity in cortical sensorimotor areas extending to the cerebello-thalamo-cortical pathway and parietal and frontal brain structures. Dopaminergic therapy normalized this increased connectivity. Notably, patients showed decreased interconnectedness in the medicated compared to the unmedicated condition, encompassing putamen, precuneus, supplementary motor and sensorimotor areas bilaterally. Similarly, lower connectivity was found comparing medicated patients to controls, overlapping with the within-group comparison in the putamen. Seed-based analyses revealed that dopaminergic therapy reduced connectivity in motor and default mode networks. Lower connectivity in the putamen correlated with longer disease duration, medication dose, and motor symptom improvement. Notably, atrophy and connectivity changes were topographically dissociated. After chronic treatment, dopaminergic therapy decreases connectivity of key motor and default mode network structures that are abnormally elevated in PD off condition.

## Introduction

Parkinson’s disease (PD) is the second most common neurodegenerative disorder^1^, but its definite cure still represents an unmet need^2^. Nevertheless, PD underlying molecular pathology, affecting the nigro-striatal dopaminergic pathway, is relatively known^3^. Accordingly, considering PD as disconnection syndrome related to histopathology (i.e. “molecular nexopathy”)^4,5^, provides a novel framework for the study of treatment-induced brain changes. In this view, brain functional connectivity assessed with magnetic resonance imaging (MRI) is a candidate biomarker to investigate both brain changes related to the PD itself and their modulation by means of different therapies.

Dopaminergic therapy (DT), based on dopamine agonists and on levodopa (3,4-dihydroxy-l-phenylalanine), restores striatal dopaminergic neurotransmission and still represents the most clinically effective treatment^6^. However, clinical improvement caused by DT is progressively reduced as the treatment, together with neurodegeneration, progresses over time. After few years, treatment efficacy decreases (i.e. wearing-off phenomenon) and several motor (e.g. dyskinesias) and non-motor (e.g. affecting mood, reward processing, and cognition) side effects can cause severe distress to the patients^7,8^. Notwithstanding, the impact of DT on the brain after several years of treatment and in patients with long disease duration has not been defined yet.

Resting-state functional MRI (rs-fMRI) has been previously implemented to investigate how DT modulates brain functional connectivity, as summarized by a recent review^9^. Overall, DT modulated the functional connectivity within the basal ganglia-thalamo-cortical network and the sensorimotor network, inducing both increases and decreases in connectivity^9^, where equivocal findings were presumably related to clinical and methodological heterogeneity. Specifically, only a modest number of studies compared the DT effect within a single PD cohort, focusing either on patients in the early-to-moderate disease stages^10–17^ or on the acute response to levodopa challenge in *de novo* PD^18,19^ (Table S4). Along the same line, a previous study from our group^20^ has shown that the acute levodopa administration in PD patients leads to functional connectivity increases in the cerebellum and brainstem, core structures in the motor network. Nevertheless, the effect of DT after several years of chronic pharmacological treatment in PD has not been investigated yet with brain rs-fMRI. To address this issue, we selected a patient cohort with longer disease duration and under chronic DT. For each PD patient, we performed two MRI sessions, one with and the other without their usual DT medications (respectively named, PD-ON and PD-OFF conditions). This study design allowed investigating connectivity changes in patients stabilized in the ON condition rather than after a single dose (see Methods section for further details). We hypothesized that DT would modulate brain functional connectivity mainly, but not only, in the motor network. However, given the exploratory nature of our research question, we preferred a conservative approach to whole-brain analysis, without constraints from *a priori* assumptions. To fulfill this aim we opted for eigenvector centrality (EC) mapping^21^, a common graph-based method for network analysis also implemented by the Google PageRank algorithm^22^. Indeed EC, at difference from other common methods for functional connectivity (e.g. seed based and principal component analysis) does not rely on specific hypotheses and does not require arbitrary decisions. In brief, ECM assigns a measure of weighted centrality to nodes (i.e. voxel) in the network, based on the assumption that more prominent nodes are linked to many other important nodes. Moreover, since ECM is a local network measure, we complemented the analysis by applying seed-based connectivity approaches to uncover specific region-to-region connectivity patterns. Between group comparisons were mainly related to PD effects in each medication condition (i.e. comparing PD-OFF with healthy controls and comparing PD-ON with healthy controls), whereas the within group comparison reflected DT effects (i.e. comparing PD-ON with PD-OFF). Finally, we also investigated the clinico-functional correlations between centrality measures and clinical variables, such as overall motor impairment, as assessed with the Unified Parkinson’s Disease Rating Scale-III (UPDRS-III), disease duration, as well as treatment dose and duration.

## Results

### EC mapping group comparison

#### Within group effect

**Figure 1:**
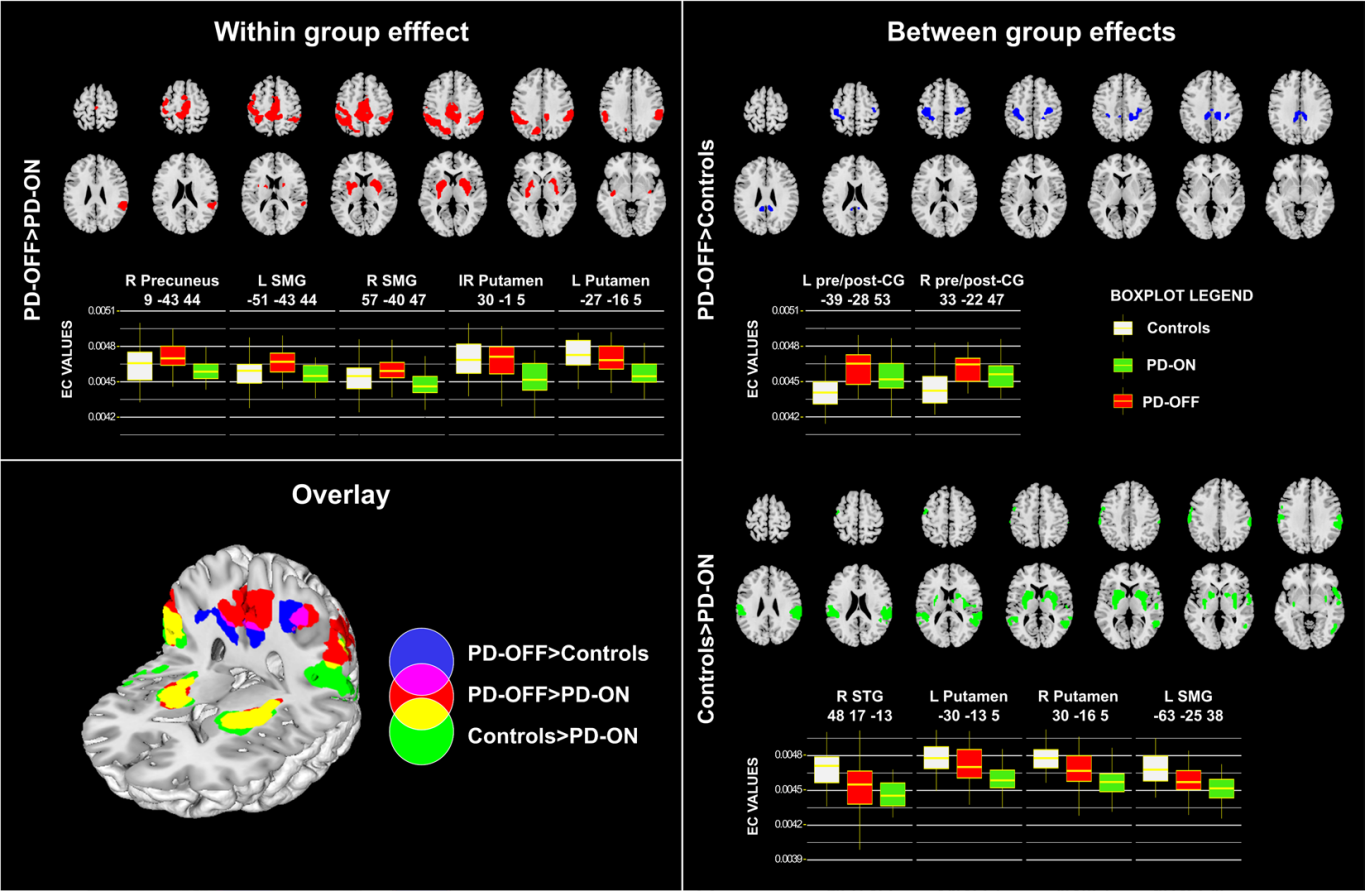
Whole-brain eigenvector centrality group comparisons. Within group effect: EC reductions in PD-ON compared to PD-OFF. Between group effects: EC increases in PD-OFF compared to controls and EC decreases in PD-ON compared to controls. 3D overlay is shown. The boxplots show EC values in the peaks of significant clusters (x, y, z MNI coordinates). p < 0.05 FWE at cluster level. Brain images displayed in neurological convention (left hemisphere on the left of the image). Abbreviations: L: left; R: right; SMG: Supramarginal Gyrus; STG: Superior Temporal Gyrus; CG: Central Gyrus. Unthresholded results for the displayed contrasts can be found on NeuroVault https://neurovault.org/collections/3976/.

Significant differences were found at the whole-brain level for the contrast PD-OFF > PD-ON, but not for the opposite. Specifically, EC reductions were detected in ON as compared to OFF condition in the bilateral putamen and globus pallidus, as well as in a large cortical cluster encompassing the precuneus, the left and right sensorimotor cortex and the supplementary motor area (Fig. 1).

#### Between group effects

Comparing PD-OFF and controls, we found a significant increase in EC in the patients in the bilateral pre- and post-central gyri and in the precuneus (Table S3). The PD-OFF < Controls analysis did not provide significant results (Fig. 1). The comparison between PD-ON and controls yielded significant results only for the Controls > PD-ON comparison. PD-ON patients showed significant reductions in EC bilaterally in the putamen, globus pallidus, and supramarginal gyri and in the left superior temporal cortex (Fig. 1).

### Correlation analysis

**Figure 2:**
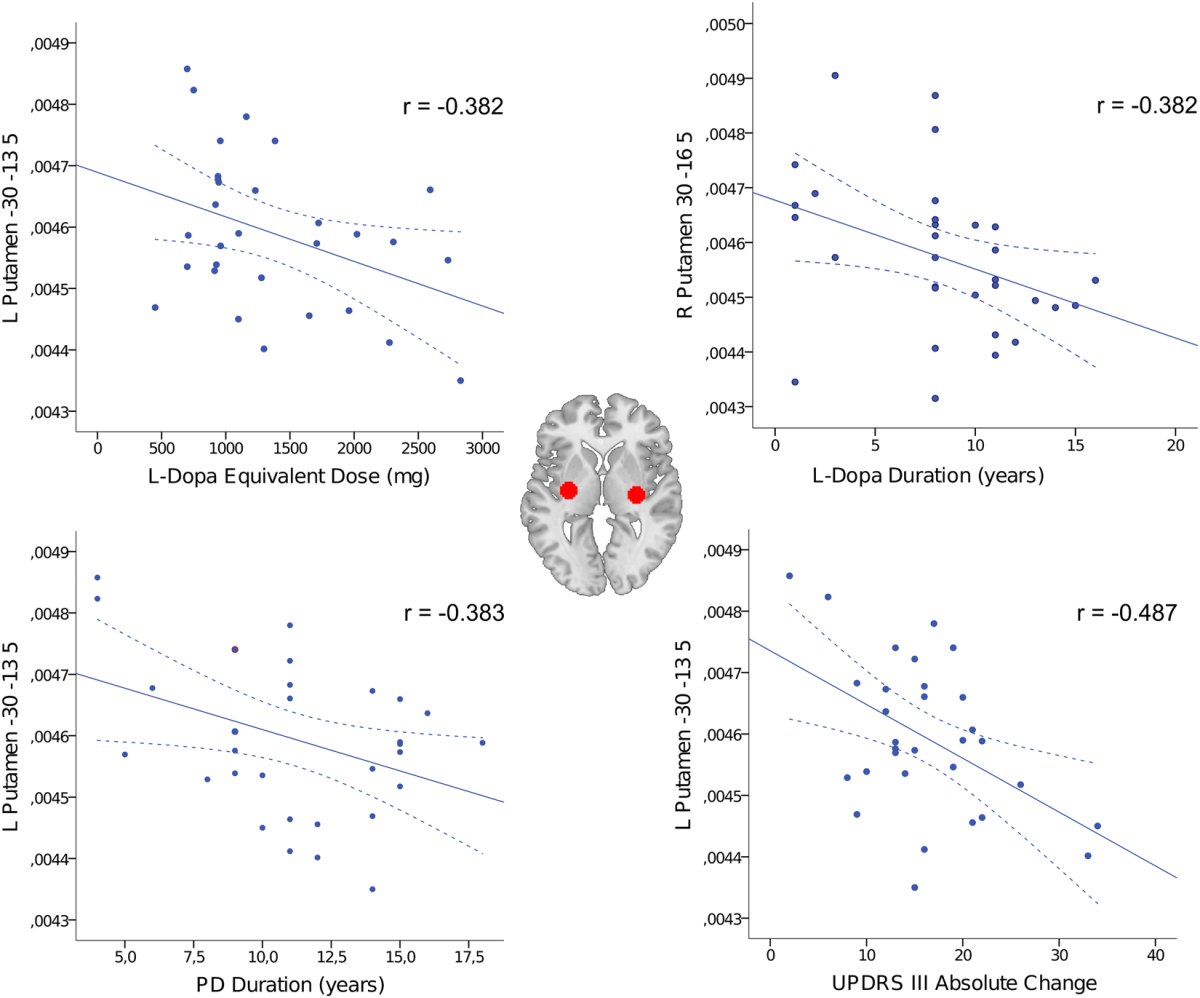
Negative correlations between EC values in PD-ON and clinical variables. Selected statistically significant (p < 0.05) results are shown. MNI coordinates and Pearson correlation coefficients (r) are reported. Putaminal seeds are displayed on the axial brain section. Solid and dashed lines represent, respectively, the regression line and the 95% confidence intervals. Abbreviations: L-Dopa: levodopa; L left; R right.

Based on the group comparison results, we investigated negative correlations between the identified EC changes and disease duration, treatment dose and duration, and UPDRS-III change between medicated and unmedicated states. In PD-ON condition, disease and levodopa duration, levodopa equivalent dose and UPDRS-III change negatively correlated with EC values. Statistically significant (p < 0.05) negative correlations were found between clinical variables and EC values in the peaks located in the left and right putamen (Fig. 2). Specifically, levodopa dose correlated with reduced EC values in the left (Montreal Neurological Institute (MNI) coordinates: x = −27, y = −16, z = 5 and −30, −13, 5) and right (30, −16, 5) putamen. Levodopa duration showed negative correlations with EC values in the right putamen (30, −16, 5), while both disease duration and UPDRS-III change in the left putamen (respectively in −27, −16, 5 and −30, −13, 5, and in −30, −13, 5).

Moreover, we correlated the UPDRS-III scores with whole-brain EC values, pooling together ON and OFF states in a single model. The correlation between the pooled (ON/OFF) UPDRS-III scores and EC values was significant in the bilateral lentiform nuclei, in the precuneus, in the bilateral sensorimotor cortex and supplementary motor area, closely resembling the pattern emerged from the within group comparison. In addition, significant correlations were found in the right superior temporal and inferior frontal gyri, in the left cerebellum and in the fusiform gyrus (Fig. S2).

### Seed-based connectivity

#### PD-OFF > Controls effect

**Figure 3:**
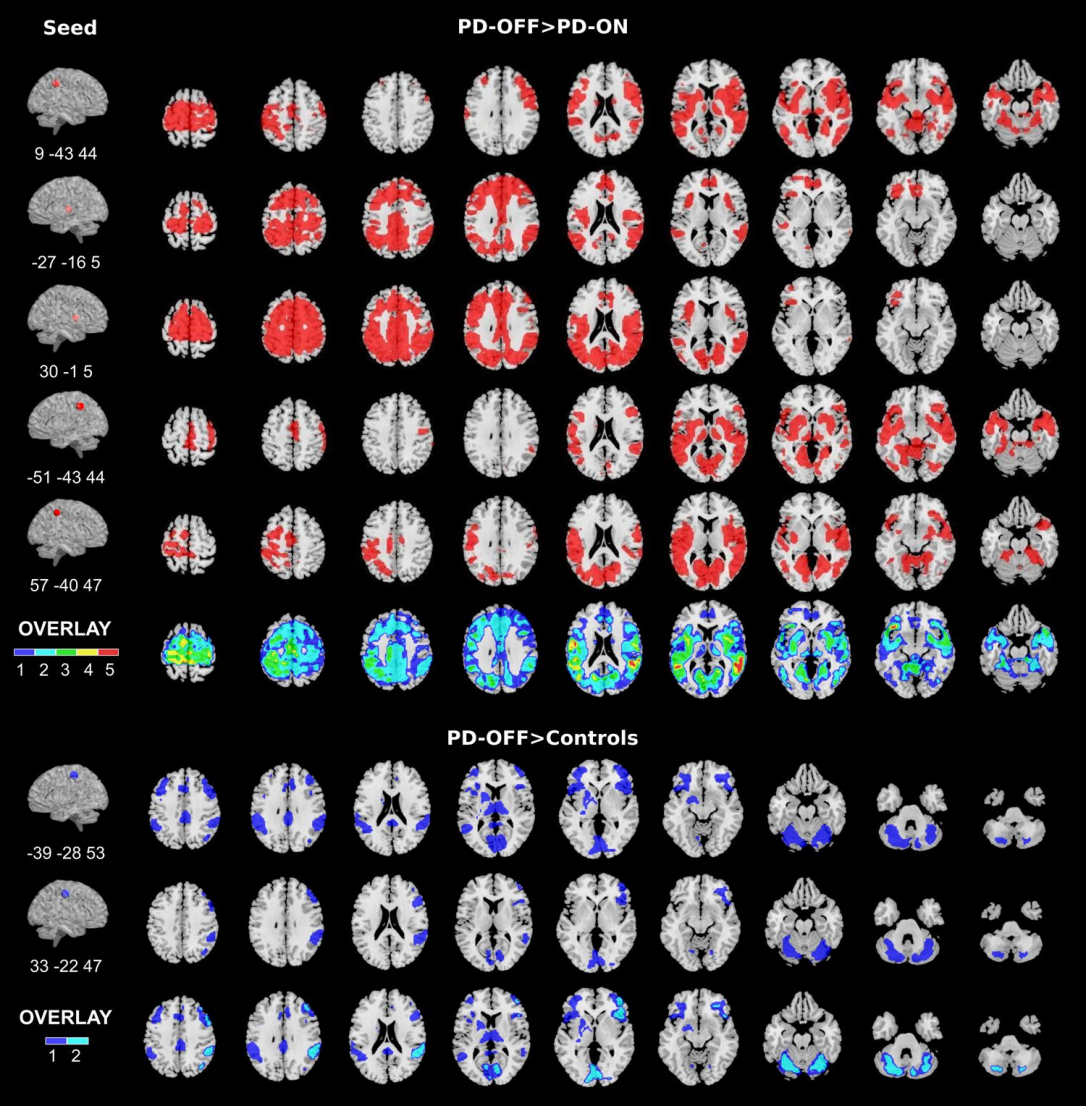
Results of seed-based connectivity analysis. On the left, the seeds are represented around the peak MNI coordinates. From top: right precuneus, left and right putamen, left and right supramarginal gyri, left and right pre-/post-central gyri. On the right, group comparisons of seed-correlation maps showing decreased connectivity within the patient group (PD-OFF > PD-ON) and increased connectivity in PD-OFF > Controls. The overlay maps display the brain regions that are more consistently associated with disease and treatment effects superimposing the results of the studied seeds. Brain images displayed in neurological convention (left hemisphere on the left of the image). p < 0.05 FWE at the cluster level. Unthresholded results for the displayed contrasts can be found on NeuroVault https://neurovault.org/collections/3976/.

This analysis (seeds in the peaks of the PD-OFF > controls difference) revealed widespread increased connectivity in the PD-OFF as compared to controls. Namely, the left cortical seed showed increased connectivity with posterior cingulum, bilateral inferior parietal cortex, bilateral thalamus, left putamen and inferior, middle frontal and orbitofrontal cortex, as well as the cerebellum (Fig. 3). Similarly, the right seed presented increased connectivity with the ipsilateral inferior parietal and inferior and middle frontal cortex, the bilateral posterior cingulum, lingual gyrus, and cerebellum bilaterally. Comparing PD-ON and controls no significant increase in connectivity was detected.

#### PD-OFF > PD-ON effect

The PD-OFF/ON comparison revealed significant results only for PD-OFF > PD-ON. Namely, the right precuneus showed connectivity decreases in ON state with several brain structures of the motor network, encompassing bilaterally the basal ganglia (particularly the putamen), the primary sensorimotor cortex, the vermis and the cerebellum. In addition, extensive connectivity reductions were found with the inferior temporal, insular and dorsolateral prefrontal cortex (more on the right) and in other segments of the precuneus, in the cuneus and lingual gyri. The bilateral putamen seeds revealed significant connectivity reductions with primary sensorimotor and premotor regions, as well as with components of the default mode network (DMN), such as the anterior cingulate and superior medial frontal cortex, the posterior cingulum and precuneus and the bilateral inferior parietal cortex (supramarginal and angular gyri). Finally, with the seeds placed in the supramarginal gyri, significant DT-induced connectivity decreases were evident in the contralateral sensorimotor and supplementary motor cortex, in the putamen and thalamus (more pronounced for the left seed), in the calcarine cortex and lingual gyrus and extensively in the temporal cortex and in the vermis. Figure 3 displays the overlay between connectivity patterns from the different seeds for PD-OFF > controls and PD-OFF > PD-ON effects, to highlight the brain regions more consistently involved.

### Voxel-based morphometry

T1-weighted MRI data were analyzed by means of voxel-based morphometry (VBM) comparing PD patients and controls. The VBM analysis revealed significant volume loss in PD compared to controls in the bilateral caudate heads and, to a less extent, in the left dorsal putamen, as well as in the cuneus and calcarine cortex, in the left middle and inferior temporal gyrus and in the medial frontal and orbitofrontal cortex. Remarkably, there was no spatial overlap, rather a regional dissociation, between functional (EC changes) and structural (atrophy) alterations (Fig. S3).

## Discussion

This study investigated the DT effect on brain connectivity in advanced PD after several years of pharmacological treatment. We found that the PD-OFF condition in comparison with controls is associated with EC increases in sensorimotor cortical regions that are related to connectivity increases with cerebellum, thalamus, basal ganglia as well as parietal and frontal structures. As opposed to this PD-OFF condition, DT mainly induced normalization of the abnormal increases and further widespread decreases in the connectivity level, especially in the bilateral lentiform nuclei, precuneus and sensorimotor cortices, and improved motor clinical symptoms.

As aforementioned, the previous within-group ON-OFF studies investigated the DT effect on rs-fMRI connectivity focusing on either *de novo* patients^18,19^ or early-to-moderate disease stages (average disease duration in previous studies: 3.9 ± (SD)1.4 years, range: 1.6–5.7)^10–17,20^. However, despite every single DT dose in PD induces an acute (i.e. short-duration) response, its prolonged use leads to a long-duration response^23,24^. This latter likely reflects dopamine-induced corticostriatal plasticity^25^, rather than the immediate response to the drug^24^. Recently, also Akram *et al*.^26^ investigated advanced PD, showing, by means of rs-fMRI and seed-based analysis, that levodopa’s clinical efficacy is associated with modulation of basal ganglia connectivity pattern in the PD-ON condition^26^. Our study provides a deeper insight into the DT effect in advanced, chronically treated PD. First, we captured the DT response when the patients were in a stable ON condition. Second, the ECM analysis^21,27^ provides a novel, data-driven perspective into DT-related connectivity changes in PD. The advantage is that ECM is not based on *a priori* assumptions, as in the case of seed-based connectivity as implemented in several previous studies^11,12,26,28^. Moreover, while seed-based analysis defines the connectivity between the selected seed and the rest of the brain, ECM assesses the interconnectedness of each node with all the other nodes in the network^21^. Finally, considering PD-ON, PD-OFF and controls, we were able to investigate both effects related to treatment and to the disease itself on brain connectivity, though their interplay makes the distinction between the two challenging.

### Between group effects

Compared to controls, we observed increased EC in sensorimotor cortical areas bilaterally and precuneus in PD-OFF, i.e. in the state that better corresponds to native PD symptoms. According to neuropathological evidence^3^, these cortical areas are the latest affected during the disease course, possibly explaining their sensitivity to functional reorganization in advanced PD. Notably, seed-based connectivity revealed several associated brain regions, ranging from the cerebellum, thalamus and basal ganglia to frontal and parietal associative cortices. At a closer look, we could group those regions as part of two networks that show aberrant increased connectivity: the cerebello-thalamo-cortical network and the DMN. On one hand, increased functional activation of the cerebello-thalamo-cortical network during motor tasks execution^29,30^ as well as enhanced functional connectivity at rest^31^ have been reported in PD as compared to controls, especially for tremor. On the other hand, mixed findings have been reported concerning DMN functional connectivity in PD, where both increases^32^ and decreases^33^ have been described, possibly due to the clinical variability of the studied cohorts. Consistently with our results, Kwak *et al*.^12^ investigated seed-based connectivity in mild-to-moderate PD and reported increases in striato-cortical connectivity in PD-OFF compared to controls. A subsequent study found increased amplitude of low-frequency fluctuations in PD-OFF compared to controls in cortical motor areas and medial and middle prefrontal cortex^13^. Of note, ECM and amplitude of low frequency fluctuations provide different results, since the former is a measure of centrality, while the latter takes into account the regional intensity/strength of spontaneous fluctuations in the BOLD signal^34^. In addition, a recent meta-analysis^35^ outlined the convergence among rs-fMRI studies comparing PD-OFF > controls, reporting hyperconnectivity in the right supramarginal gyrus and bilaterally in the inferior parietal lobules. On the contrary, no consistent findings emerged for the PD-OFF < controls comparison^35^. This result aligns with our study and stresses once more the need to refocus the attention on brain regions outside the core motor network that have been so far neglected. Notably, as pointed out also by Kwak *et al*.^12^, the increases in connectivity are in line with the observed increased synchronicity in corticostriatal networks in dopamine depleted conditions^36,37^, including PD^38^. Nevertheless, despite functional connectivity increases in OFF conditions have been consistently reported^12,13,31,32^, whether they represent purely pathological signatures or compensatory mechanisms is still a matter of debate^39^.

At difference with our findings, previous studies^14,16–19,28^ also reported connectivity reductions in the PD-OFF < controls comparison. In particular, Lou *et al*.^40^ compared non-depressed PD-OFF and healthy controls using voxel-wise ECM as in our study. At difference with our results, they reported reduced interconnectedness in PD-OFF as compared to controls in several cortical regions^40^. However, their study used a withdrawal time for the medication of 12 hours, without controlling for the kinds and doses of antiparkinsonian drugs administered to the patients. If DT included a dopamine agonist, 12 hours would not be enough to wash-out the medication^24^, thus the reported connectivity reductions might still be influenced by the ON medication condition. In our study, the time between DT withdrawal and OFF MRI was chosen to maximize the drug washout while keeping the patients in the uncomfortable OFF state for the shortest time possible. However, it has to be considered that the long-duration response induced by chronic DT slowly decreases over time after treatment discontinuation^24^, thus possibly masking, also in our study, the complete range of disease-related connectivity changes. Moreover, another factor that might influence our results is that subtle connectivity reductions in subcortical structures (e.g. substantia nigra and ventral tegmental area) are difficult to capture at the whole-brain level due to the conservative significance threshold. Consistently with our results, the meta-analysis of the rs-fMRI literature concerning the PD-ON < controls comparison showed a convergent connectivity reduction in the right inferior parietal lobule in PD-ON^35^. Remarkably, we found decreased interconnectedness in the PD-ON state that goes beyond the normal physiological level of controls. Since we observed convergent connectivity reductions comparing PD-ON with both controls and PD-OFF, we believe that these phenomena are mainly treatment-related, strongly supporting a DT-induced change in resting-state connectivity. However, this does not exclude that the basal ganglia, affected by underlying pathological process, over-react to DT, possibly explaining the exaggerated connectivity decrease observed in the PD-ON < controls comparison.

### Within group effect

DT reduced EC in the precuneus, sensorimotor cortex and in the lentiform nuclei. Notably, the negative correlations in the putamen between EC and UPDRS-III improvement suggested that the DT effect is associated with a better treatment efficacy. Moreover, the association between treatment duration and dose and connectivity reductions supported the idea that putaminal changes reflect chronic DT effects. Indeed, it has been shown that long-duration DT effects are built up over time and are dose-dependent^41^. The positive correlation between EC and UPDRS-III (i.e. the higher the connectivity, the worse the motor performance) provided complementary results to the ON-OFF group comparison, taking into account the individual variability in UPDRS-III change. Previous within-subject rs-fMRI studies on DT in PD are heterogeneous concerning imaging analysis methods and clinical features of the studied cohorts, thus reporting mixed findings^10–19,28^. Overall, DT reverted the functional connectivity abnormalities of the OFF state, thus having a normalizing effect^9^. Among previous studies, only one applied a similar methodological approach, studying brain interconnectedness by means of degree centrality, an unweighted version of ECM^17^. However, degree centrality was estimated only for a small set of predefined regions of interest in the motor network and not voxel-wise, thus hampering comparability with our results. As aforementioned, Kwak *et al*.^12,13^ reported abnormally increased connectivity in PD-OFF compared to controls and, accordingly, levodopa decreased functional connectivity, in particular inside the motor network. Similarly, we showed that DT suppresses abnormally elevated EC. However, we cannot say if this decrease corresponds to the turn-off of a pathological mechanism or of a compensatory functional reorganization that is no longer needed in ON state. The seed-based analysis confirmed widespread connectivity reductions in PD-ON compared to PD-OFF, mainly encompassing brain structures in the cerebello-thalamo-cortical network and DMN. For example, connectivity decreased in PD-ON between the precuneus, as DMN hub, and primary sensorimotor cortex, cerebellum and putamen, key structures of the motor network. Likewise, putaminal connectivity with the medial frontal and anterior cingulate cortex, posterior precuneus and cingulum and parietal associative cortices was reduced, mimicking the DMN architecture. Consistently, Kelly *et al*.^42^ demonstrated that levodopa induces brain functional connectivity decreases in young healthy subjects between basal ganglia and DMN and within the DMN itself^42^. This supports once again the DMN relevance in PD and its role as treatment target.

When discussing both between and within group effects in comparison to the previous literature, it has to be taken into consideration that there is a notable heterogeneity in the methods implemented for the analysis of rs-fMRI data (see for example Table S4). This represents a limit, since different methods, often based on specific assumptions, provide different perspectives on the topic of brain functional connectivity.

#### Voxel-based morphometry

VBM revealed PD-related atrophy, affecting both motor and non-motor areas. A recent review reported mixed findings concerning brain atrophy in PD^43^. Indeed, structural changes tend to be minimal and undetectable with conventional MRI in the early stages, while, later on, heterogeneous volume decreases and cortical thinning have been observed both inside (e.g. striatum and substantia nigra) and outside the motor network (e.g. olfactory bulb, orbitofrontal cortex, occipito-parietal cortex, frontal lobe, hippocampus and anterior cingulum)^43^. Notably, in our study, VBM and ECM changes were spatially dissociated, suggesting that both disease- and treatment-related connectivity changes are localized in brain regions spared by advanced neurodegeneration.

In this study, we investigated brain functional connectivity based on the blood-oxygen level dependent (BOLD) signal in the rs-fMRI. The BOLD signal is tightly related to neurovascular coupling, and is influenced by changes in cerebral blood volume/flow, and oxygen consumption^44^. Previous evidence has shown that neurovascular coupling can be altered in disease states, such as hypertension, stroke and Alzheimer’s disease, and by several drugs^45,46^. However, according to recent research, neither PD, nor levodopa seem to have a significant impact on vasoreactivity and neurovascular coupling^47–49^. Accordingly, one can exclude a potential significant confound on our results when comparing patients and controls, as well as PD patients before and after treatment.

Our results represent a first insight into the effect of DT in chronically treated PD. We are aware that the interpretation of our results for the between and within group comparisons, respectively in terms of disease- and treatment-related effects would be an oversimplification, since the two processes are tightly tangled. This issue delineates the need for future longitudinal studies. We believe that a deeper insight into DT-induced neuroplasticity will pave the way to better understanding therapeutic DT effects itself and associated side effects and will contribute to set up novel therapeutic approaches. We also support the study of rs-fMRI connectivity, framing PD in the context of molecular nexopathies^4,5^, as a useful biomarker to monitor DT-related brain changes.

## Methods

### Participants

Thirty-one patients diagnosed with sporadic PD and 30 matched healthy controls were included. PD was diagnosed by neurologists specialized in movement disorders according to validated criteria^50^ (Table 1). The average (±SD) disease duration was 11.19 ± 3.56 years. Other inclusion criteria were: PD onset after 45 years of age and Hoehn and Yahr stage from I to III. In addition, stable medication at least 4 weeks before enrollment, consisting of levodopa in monotherapy or in combination with dopamine agonists (pramipexole or ropinirole). The daily levodopa equivalent dose was computed for each patient^51^. Exclusion criteria were: actual or past psychotic symptoms, antipsychotic treatment, cognitive impairment (Montreal Cognitive Assessment – MoCA – below −1.5 SD compared to Czech norms)^52^, deep brain stimulation or jejunal levodopa infusion, any illness potentially affecting motor or cognitive state and any contraindication for MRI. Individual T1, T2, and fluid-attenuated inversion recovery (FLAIR) images were inspected for obvious abnormalities, such as severe atrophy or focal lesions exceeding the normal range commonly seen in healthy subjects for that particular age as judged by experienced radiologist. As expected, given the long treatment duration, the majority of patients (26/31) experienced treatment-related complications, but mainly of mild intensity (see for details Table 1 and UPDRS-IV in Supplementary Table S1). All subjects gave their informed consent to participate in the study that was approved by the Ethics Committee of the General University Hospital in Prague, Czech Republic, in accordance with the Declaration of Helsinki.

**Table 1:** Descriptive and clinical characteristic of Parkinson’s disease and healthy control groups.

	Parkinson’s Disease	Healthy Controls	p
N	31	30	—
Age (years)	64.74 ± 7.76	63.57 ± 8.09	0.565
Gender (m/f)	18/13	15/15	0.611
Education (years)	14.10 ± 2.95	14.90 ± 3.56	0.353
Body mass index (kg/m^2^)	25.09 ± 4.65	26.88 ± 4.47	0.133
Disease duration (years)	11.19 ± 3.56	—	—
Levodopa duration (years)	8.28 ± 4.44	—	—
Levodopa equivalent dose (mg)	1371.79 ± 657.78	—	—
Levodopa complication duration (years)	4.27 ± 2.64	—	—
Dyskinesia (yes/no)	20/11	—	—
Akinesia (yes/no)	8/23	—	—
OFF Freezing (yes/no)	10/21	—	—
Scan Order (ON-OFF/OFF-ON)	16/15	—	—
UPDRS-III OFF	30.9 ± 10.16	—	<0.001*
UPDRS-III ON	14.74 ± 7.46	—	
UPDRS-III Change	16.16 ± 6.96	—	—

*Significat p-value for the difference in UPDRS-III scores between ON and OFF conditions.

### Image acquisition and study design

T1-weighted (3D- magnetization-prepared rapid gradient-echo – MPRAGE – sequence with TR = 2.2 sec, TE = 2.43 ms, inversion time = 900 ms, voxel size 1.03*1.03*1 mm, matrix = 224 × 224 and flip angle = 8 deg) and rs-fMRI data (T2*-weighted gradient-echo-planar imaging: TR = 2000 ms, TE = 30 ms, flip angle = 90 deg, voxel size 3*3*3.45 mm, 300 repetitions) were acquired on a 3 T MAGNETOM Skyra MRI (Siemens, Erlangen, Germany) at the Radiology Department, Na Homolce Hospital in Prague. A transmitter body coil and a receiver head coil with 32-channels were used. Two MRI sessions were performed in the PD cohort, namely during the unmedicated and DT states (respectively, PD-OFF and PD-ON). PD-ON patients were under their clinically optimal DT regimen to capture their typical treatment state. For PD-OFF, dopamine agonists were withdrawn 72 hours before the MRI session and stepwise replaced by levodopa to maintain clinical efficacy. Then, levodopa was withdrawn the night before the OFF MRI. If patient was on catechol-O-methyl transferase (COMT) inhibitors, they were withdrawn together with levodopa. Although some patients were additionally treated with monoamine oxidase-B (MAOB), amantadine and antidepressants, all these medications were kept constant during the withdrawal procedure to limit their influence on the DT ON-OFF comparison. The UPDRS-III assessed motor impairment in both PD-ON and PD-OFF. The sub-scores for akinesia, rigidity and tremor were computed (Table S1). PD-ON and PD-OFF MRI sessions were randomized across the patients to avoid confounding order effects.

### MRI preprocessing

Rs-fMRI images were preprocessed in SPM12 (rev.12.6685) (Wellcome Department of Imaging Neuroscience, London, UK) running on Matlab™ 9.0 (R2016a). Realignment, slice-time correction and the unified segmentation approach were implemented^53^. Eight millimeter smoothing was applied with a Gaussian kernel to increase the signal-to-noise ratio. Given the concerns regarding motion artifacts in PD patients, we carefully checked the amount of head motion during MRI for each participant. Namely, mean and maximal framewise displacement were assessed^54^. Group comparisons were run to compare PD-ON to PD-OFF and both to controls (respectively, paired-sample and two-sample t-tests). No significant group differences emerged and, notably, mean and maximal framewise displacement did not exceed the isotropic voxel size (Table S2 and Fig. S1). This suggested reliability of rs-fMRI signal changes over the time-course. However, we applied a conservative approach including motion parameters in a nuisance regression model together with the mean intensity signals extracted from the anatomical segmentation of white matter and cerebrospinal fluid for each subject. Linear drifts and noise in low-frequency signals were removed through high-pass filtering.

### Image analysis

#### Eigenvector centrality mapping

EC mapping^21^ was applied with the fastECM approach^27^. As previously introduced, EC identifies functional brain hubs, namely brain structures with high connectivity that are in turn linked to densely connected structures. Accordingly, the importance of a node in the network depends on its connections to other prominent nodes. FastECM considers both positive and negative correlations between brain regions. Since the adjacency matrix for EC computation must be symmetric and positive in order to obtain a unique largest eigenvalue (Perron-Frobenius theorem), the algorithm remaps the correlation matrix to positive values by adding 1 to all correlations. The analysis was performed at the whole-brain level using a mask created from the patients scans to avoid possible artifacts due to disease-related brain alterations. FastECM provided for each participant a 3D image containing voxel-wise EC values.

#### Group comparisons

EC maps were compared between PD-OFF and PD-ON conditions in a whole-brain paired-sample t-test (within group effect). Patients and controls were compared using an independent sample t-test with age and gender as nuisance covariates (PD-OFF vs. controls and PD-ON vs. controls, between group effects). Only results in the grey matter were considered significant applying a cluster level family-wise-error (FWE) correction for multiple comparisons (p < 0.05) after a voxel-level significance threshold at p < 0.005 (cluster extent k = 10).

#### Correlations

Based on the group comparison results (see results) we hypothesized negative correlations between EC values in the PD-ON condition and the following clinical variables: disease duration, levodopa duration, levodopa equivalent dose and UPDRS-III change (i.e. the less the connectivity, the longer the disease and treatment duration, the higher the levodopa equivalent dose and the motor improvement). We computed one-tailed correlations in IBM® SPSS® (Release 22.0.0.1) between EC values in the peaks of the group comparisons for treatment effect (PD-OFF > PD-ON, Controls > PD-ON; Table S3) and the clinical variables.

Moreover, a whole-brain correlation between UPDRS-III total score and ECM maps was run, pooling together PD-ON and PD-OFF scans in a single model. In this design, each patient is represented twice to take into account the between-subject variability in the UPDRS-III ON-OFF changes. Accordingly, a factor was specified for each subject to compensate for the repeated representation. This model provides complementary information to the group comparison analysis.

#### Seed-based connectivity

As aforementioned, EC mapping is a local network measure. We thus also applied seed-based connectivity analysis to investigate selective region-to-region connectivity changes. Two sets of seeds were selected: one in the peaks of the PD-OFF > controls comparison, and the other in the peaks of the PD-OFF > PD-ON comparison. The seeds for the PD-OFF > controls were located in the bilateral postcentral gyri (Montreal Neurological Institute – MNI – coordinates x, y, z: 33, −22, 47 and −39, −28, 57), while those for PD-OFF > PD-ON effects in the right precuneus (MNI: 9, −43, 44), bilateral supramarginal gyri (MNI: −51, −43, 44 and 57, −40, 47) and bilateral putamen (MNI: −27, −16, 5 and 30, −1, 5). Single voxels were used as seeds given that the 8 mm smoothing had already merged part of the neighbouring signal intensities. The time-courses were extracted from the seeds for each functional image and voxel-wise correlations were computed in Matlab™ 9.0. The correlation maps were used to further investigate the observed EC changes through the following comparisons: PD-OFF > PD-ON and PD-OFF > controls. As for the EC analysis, the significance level was set at p < 0.05 FWE at cluster level (cluster forming voxel-wise threshold, p < 0.005, k = 10).

#### Voxel-based morphometry

A voxel-based morphometry (VBM) analysis^55^ was run with the Computational Anatomy Toolbox (CAT-12) to investigate the topographical relationship between grey matter density and EC changes (conjunction analysis). T1-MRI data were normalized, segmented and smoothed (8 mm) before entering the statistical comparison between patients and controls. Total intracranial volume, age, and gender were modeled as covariates. As before, we selected only clusters surviving the p < 0.05 FWE correction (voxel-wise threshold: p < 0.005, k = 10).

Unthresholded results for the analyzed contrasts displayed in Figs 1, 3 and S3 can be visualized and downloaded on NeuroVault^56^ at the following link: https://neurovault.org/collections/3976/.

## Electronic supplementary material

Supplementary figures and tables

## Data Availability

The datasets analyzed during the current study are available from the corresponding author on reasonable request. All the raw data have been anonymized: personal meta-data removed from the image headers, T1-weighted data de-faced and rs-fMRI images preprocessed. The data will be shared in agreement with the European General Data Protection Regulation (GDPR). Unthresholded results for the analyzed contrasts displayed in Figs 1, 3 and S3 can be visualized and downloaded on NeuroVault at the following link: https://neurovault.org/collections/3976/.
