# Supplementary material for: Unraveling connectivity changes due to dopaminergic therapy in chronically treated Parkinson’s disease patients
Source: Sci Rep. 2018 Sep 25;8:14328. doi: 10.1038/s41598-018-31988-0 (PMC6156510; doi:10.1038/s41598-018-31988-0)
Supplement: Supplementary file 1 — Supplementary figures and tables [file 41598_2018_31988_MOESM1_ESM.pdf]

Supplementary information

Unraveling connectivity changes due to dopaminergic therapy in chronically treated Parkinson's disease patients

Tommaso Ballarini, MSc, [1] Filip Růžička, MD, Phd, [2] Ondrej Bezdicek, MA, PhD, [2] Evžen Růžička, MD, DSc, FCMA, FEAN, [2] Jan Roth, MD, PhD, [2] Arno Villringer, MD, PhD, [1,3] Josef Vymazal, MD, DSc, [4] Karsten Mueller, PhD, [1]* Matthias L Schroeter, MD, PhD, MA [1,3,5†], Robert Jech, MD, PhD [2†*]

[1] Max-Planck Institute for Human Cognitive and Brain Sciences, Leipzig, Germany

[2] Department of Neurology, Charles University in Prague, First Faculty of Medicine, Prague, Czech Republic

[3] Clinic for Cognitive Neurology, University Clinic, Leipzig, Germany

[4] Department of Radiology, Na Homolce Hospital, Prague, Czech Republic

[5] FTLD Consortium, Ulm, Germany

†Senior authors contributed equally to the study.

*Corresponding author:

Prof. Robert Jech, MD, PhD
Center for interventional therapy of movement disorders, Department of Neurology, Charles University, 1st Faculty of Medicine and General University Hospital in Prague
Kateřinská 30, 120 00 Praha, Czech Republic
Tel: +420 224965556
E-mail: jech@cesnet.cz

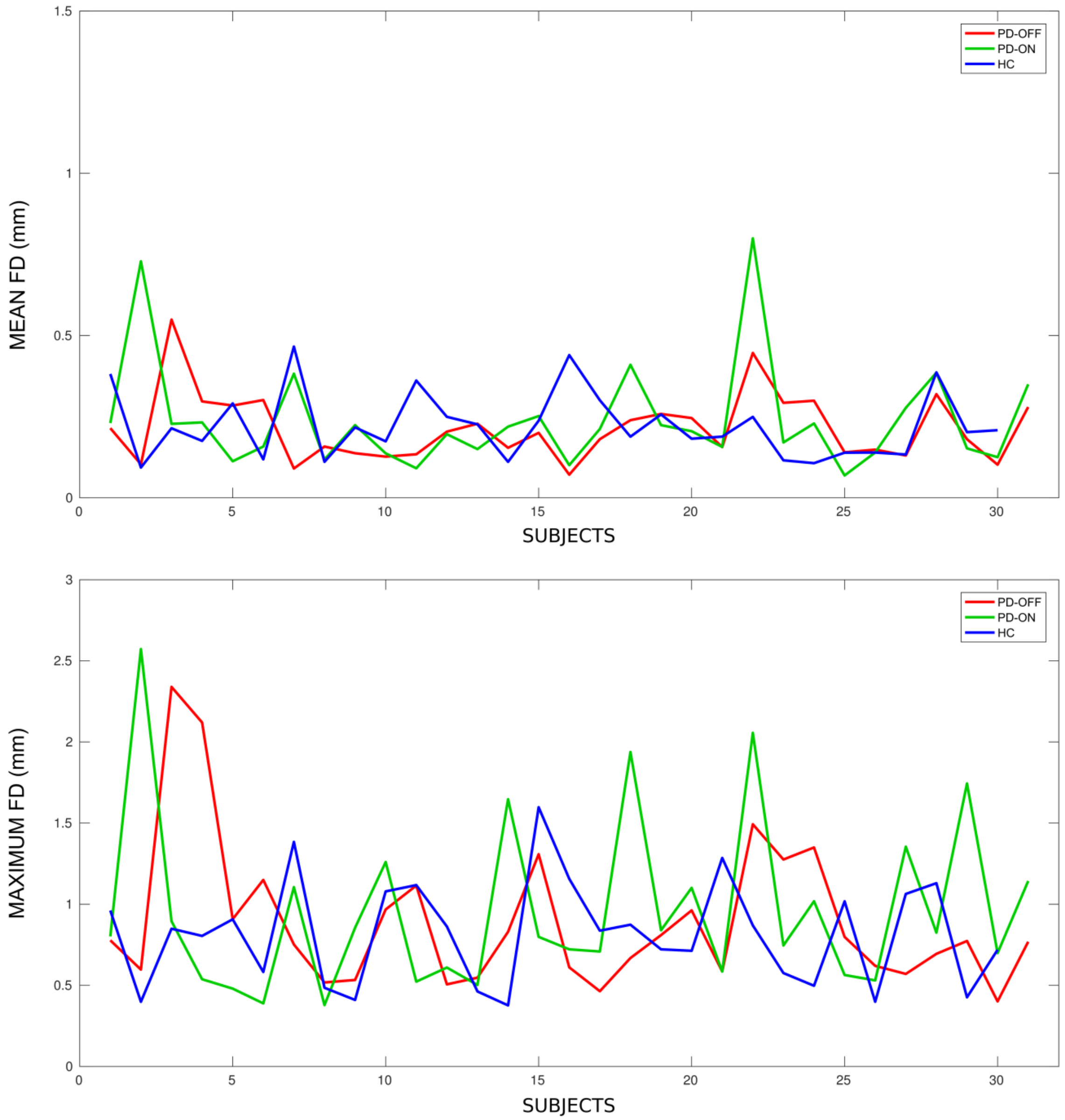

Fig. S1. Framewise displacement analysis.

Graphs showing the distributions of mean (upper row) and maximum (bottom row) framewise displacement (FD) for each Parkinson's disease patient in the two treatment conditions (green = ON and red = OFF) and for healthy controls (blue).

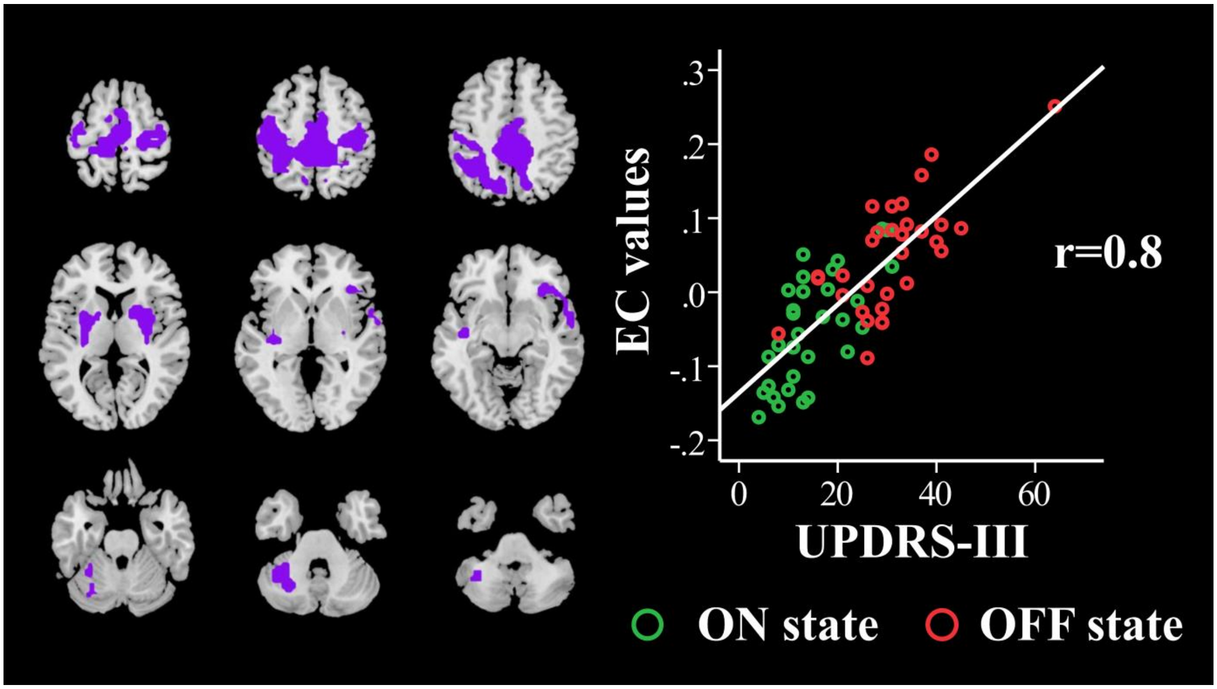

Fig. S2. UPDRS-III correlation. Correlation between UPDRS-III score pooling ON/OFF conditions and EC measures. The scatter-plot represents EC values from the peak of the correlation in the right precuneus (MNI x,y,z: 9,-46,47). Brain images displayed in neurological convention (left hemisphere on the left). p< 0.05 FWE at cluster level.

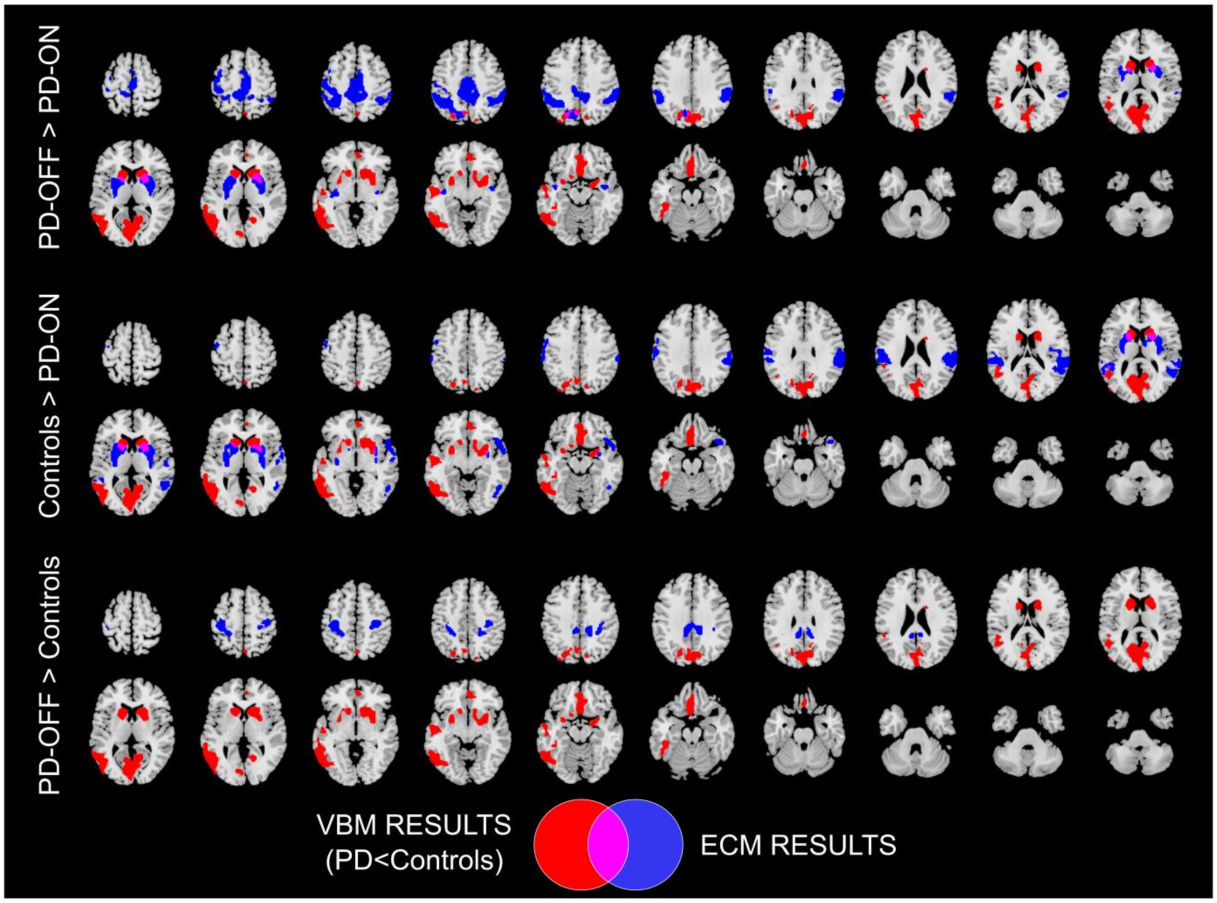

Fig. S3. Brain atrophy and functional connectivity dissociation. Overlap between PD-related brain atrophy (red) and the different group comparison results from Eigenvector Centrality Mapping (blue) as shown in Fig.1. Only minimal overlap is identified at the border between the caudate head and the putamen in the PD-OFF>PD-ON and HC>PD-ON comparisons. Brain atrophy and both disease- and treatment-related connectivity changes are overall spatially dissociated. Brain images displayed in neurological convention (left hemisphere on the left). $p<0.05$ FWE corrected at the cluster level (uncorrected whole-brain threshold: $p<0.005$, $k=10$).

Table S1 Sub-scores of the Unified Parkinson's Disease Rating Scale (UPDRS)

	ON			OFF			OFF-ON Change			P	Test Range
	Mean±St. Dev.	Min	Max	Mean±St. Dev.	Min	Max	Mean±St. Dev.	Min	Max		
**UPDRS-I**	1.48 ± 1.77	0	7	1.58 ± 1.75	0	7	0.09 ± 0.3 †	0	1	0.82	0-16
**UPDRS-II**	7.58 ± 4.92	0	17	13.23 ± 6.44	3	31	5.68 ± 5.03	0	16	<0.001*	0-52
**UPDRS-III**	14.71 ± 7.48	4	31	30.87 ± 10.12	8	64	16.16 ± 6.91	2	33	<0.001*	0-108
Tremor	0.93 ± 1.15	0	5	3.13 ± 3.48	0	16	2.19 ± 3.69	-2	16	<0.001*	0-28
Rigidity	2.13 ± 2.16	0	7	6.52 ± 3.17	0	12	4.38 ± 2.77	0	11	<0.001*	0-20
Akinesia	7.45 ± 4.02	2	17	12.55 ± 4.05	5	21	5.10 ± 3.36	-1	14	<0.001*	0-63
Axial Symptoms	4.19 ± 2.89	0	11	8.68 ± 4.78	1	20	4.48 ± 2.93	1	12	<0.001*	0-42
**UPDRS-IV**	4.55 ± 3.62							0	13	-	0-23
Dyskinesias	1.68 ± 1.89							0	7	-	0-13
Clinical Fluctuations	2.13 ± 1.76							0	5	-	0-7
Other complications	0.71 ± 0.86							0	3	-	0-3

† In 28 patients there is no change in UPDRS I between ON and OFF state
*p-value from 2-sample t-test comparing ON and OFF scores
Abbreviations: UPDRS: Unified Parkinson´s Disease Rating Scale

Table S2. Group comparison between mean and maximum framewise displacement values.

	Mean Framewise Displacement			Maximal Framewise Displacement		
	PD-ON vs PD-OFF	PD-ON vs HC	PD-OFF vs HC	PD-ON vs PD-OFF	PD-ON vs HC	PD-OFF vs HC
**t(df)**	0.84 (30)	0.53 (59)	0.26 (59)	0.54 (30)	1.29 (59)	0.77 (59)
**p**	0.41†	0.59‡	0.79‡	0.59†	0.20‡	0.44‡

† paired-sample t-test
‡ two-independent sample t-test
Abbreviations: PD-ON medicated Parkinson´s disease patients; PD-OFF OFF medication Parkinson's disease patients; HC Healthy Controls

Table S3. Spatial localization of the significant clusters in the whole-brain ECM group comparisons.

	t	$P_{FWE\ (cluster\text{-}level)}$	x y z	Hemisphere	Cluster size	Cluster Label
**PD-OFF>PD-ON**	5.53	0.000	9 -43 44	R	681	Precuneus
	5.17	0.000	-51 -43 44	L	598	Supramarginal Gyrus
	4.98	0.001	57 -40 47	R	375	Supramarginal Gyrus
	4.79	0.011	30 -1 5	R	243	Putamen + Pallidum
	4.47	0.013	-27 -16 5	L	237	Putamen + Pallidum
**HC>PD-ON**	5.32	0.037	48 17 -13	R	184	Temporal Pole
	5.28	0.007	-30 -13 5	L	267	Putamen + Pallidum
	5.19	0.000	30 -16 5	R	788	Putamen + Pallidum
	4.85	0.003	-63 -25 38	L	304	Supramarginal Gyrus
**PD-OFF>HC**	4.66	0.078 (0.042 P_{FDR})*	-39 -28 53	L	150	Pre-Post Central Gyrus
	4.19	0.003	33 -22 47	R	299	Pre-Post Central Gyrus

Abbreviations: PD-ON medicated Parkinson´s disease patients; PD-OFF OFF state Parkinson's disease patients; HC Healthy Controls; L Left; R Right; FWE Family Wise Error

*The significance for the left cortical cluster was close to the FWE threshold (p=0.078 FWE, p=0.042 FDR) and we regarded it as a true finding due to its symmetry to the right one.

Table S4. Review of previous studies investigating the dopaminergic therapy effect (ON-OFF difference) on brain resting-state functional connectivity.

**Authors (year)**	**PD Stage (mean±SD years)**	**N**	**Connectivity method**	**Type of analysis**	
				**Whole-Brain**	**ROI**
Wu et al. (2012)	Early (2.05 ± 0.64) de novo PD	16	Effective Connectivity (Granger causality)		X
Esposito et al. (2013)	Early (1.6 ± 0.51) de novo PD	20 (10 Levodopa 10 Placebo)	ICA	X	X
Szewczyk-Krolikowski et al. (2014)	Moderate (2.55 ± 0.98)	19	ICA	X	
Yang et al (2016)	Early (3.76±2.79)	22	Seed-based		X (seed)
Gao et al. (2016)	Early (4.9 + 2.6)	30	Seed-based		X (seed)
Wu, Wang et al. (2009a)	Early (4.1 ± 1.8)	22	Graph (degree connectivity)		X
Wu, Long et al. (2009b)	Early (4.1 ± 1.8)	22	ReHo	X	
Kwak et al. (2010)	Mild to moderate (5.2 ± 3.4)	25	Seed-based		X (seed)
Kwak et al. (2012)	Moderate (5.4 ± 3)	24	ALFF	X	X
Berman et al. (2016)	Moderate (5.73 ± 3.45)	19	Graph (network topology)	X (ROI-based parcellation)	
Vo et al. (2016)	Not reported	8	ICA	X	
Present study	Intermediate to long (11.19 ± 3.56)	31	Eigenvector centrality and Seed-based	X	X (seed)

Abbreviations: ICA Independent Component Analysis; ReHo Regional Homogeneity; ALFF Amplitude of Low Frequency Fluctuations; ROI Region Of Interest
